# An Improved YOLOv7-Based Model for Real-Time Meter Reading with PConv and Attention Mechanisms

**DOI:** 10.3390/s24113549

**Published:** 2024-05-31

**Authors:** Xiancheng Peng, Yangzhuo Chen, Xiaowen Cai, Jun Liu

**Affiliations:** 1 Artificial Intelligence, Xiangtan University, Xiangtan 411100, China; 202105570214@smail.xtu.edu.cn (X.P.); 202105570211@smail.xtu.edu.cn (J.L.); 2 School of Automation and Electronic Information, Xiangtan University, Xiangtan 411100, China; chenyz5@xtu.edu.cn

**Keywords:** YOLOv7, meter-sensor reading, real-time detection, deep learning

## Abstract

With the increasing complexity of the grid meter dial, precise feature extraction is becoming more and more difficult. Many automatic recognition solutions have been proposed for grid meter readings. However, traditional inspection methods cannot guarantee detection accuracy in complex environments. So, deep-learning methods are combined with grid meter recognition. Existing recognition systems that utilize segmentation models exhibit very high computation. It is challenging to ensure high real-time performance in edge computing devices. Therefore, an improved meter recognition model based on YOLOv7 is proposed in this paper. Partial convolution (PConv) is introduced into YOLOv7 to create a lighter network. Different PConv introduction locations on the base module have been used in order to find the optimal approach for reducing the parameters and floating point of operations (FLOPs). Meanwhile, the dynamic head (DyHead) module is utilized to enhance the attention mechanism for the YOLOv7 model. It can improve the detection accuracy of striped objects. As a result, this paper achieves ${\mathrm{mAP}}_{50}^{val}$ of 97.87% and ${\mathrm{mAP}}_{50:90}^{val}$ of 62.4% with only 5.37 M parameters. The improved model’s inference speed can reach 108 frames per second (FPS). It enables detection accuracy that can reach ±0.1 degrees in the grid meter.

## 1. Introduction

As grid industrial automation advances, intelligent inspections are repeatedly propelled into the spotlight. Automating the inspection of various old-fashioned instruments is challenging in grid meter detection. A single medium-sized substation may be equipped with as many as 800 to 1000 pointer-type meters. High-frequency manual inspections inevitably result in an increased error rate. Therefore, it is imperative to propose an effective and intelligent solution to alleviate this repetitive and burdensome work.

Currently, there has been much research in automatic grid meter recognition. In the past, traditional computer vision and digital image processing were commonly used for reading meter data. Zhang et al. [1] improved the visual saliency of pointer meter regions by establishing the localized pixel inhomogeneity factor (LPIF) model. And, Hough transform [2] was utilized to recognize the meter readings. Meng et al. [3] employed segmentation of pointers through thresholding and the area growth method. They used the Bresenham algorithm to calculate the deflection angle between the center line of the pointer and the zero scale. This method incorporates dynamic thresholding and pixel restoration mechanisms into the recognition process. This can cause it to be robust. However, in some environments with significant lighting changes, reflections of the meter dial face can affect the recognition of the pointer features.

Because of the proliferation of deep learning, numerous methods have emerged. He et al. [4] utilized the Mask R-CNN [5] for grid meter detection. Target detection was employed to locate the dial, followed by semantic segmentation to delineate the pointer region. Principal Component Analysis (PCA) was used to fit the slope of the pointer, and accurate meter readings were achieved. Advanced deep-learning techniques were used in this method for accurate detection. However, the high computing overhead of the semantic segmentation model results in it being difficult to deploy on edge devices, resulting in a low real-time performance. Fan et al. [6] improved YOLOv5 based on the Global Context (GC) [7] module. The meter was located through target detection. The U-net [8] was employed for pointer segmentation and keypoint detection. They also used PCA to fit the slope of the pointer. This approach can adapt to a certain level of complexity in the environment. However, it requires two separate model inferences—the YOLOv5 target detection and U-net semantic segmentation. It results in significant computational overhead and longer processing time. Therefore, a grid pointer recognizer that combines high accuracy and robustness with real-time performance is still needed.

Regarding the problem mentioned above, an improved model based on YOLOv7 [9] is implemented that combines Partial Convolution (PConv) [10] and Dynamic Head (DyHead) [11] for grid meter detection. Additionally, an adaptive calibration algorithm has been incorporated for pointer reading calculation. This paper achieves relatively accurate readings in complex environments while maintaining a high real-time performance and lower parameters. Meanwhile, it demonstrates stable recognition when detecting pointers in dark, blurry, and small target scenarios.

**Figure 1:**
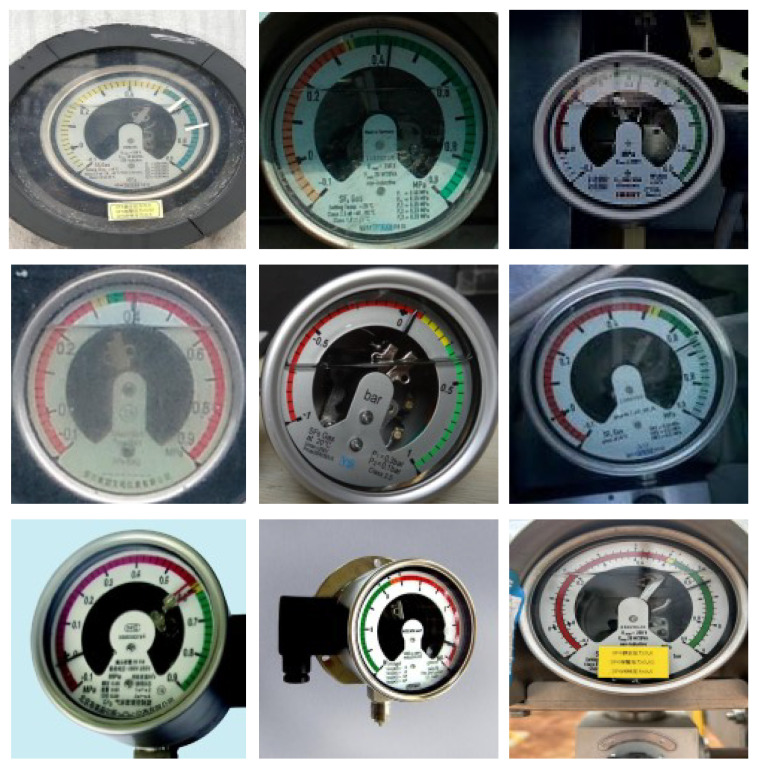
Overview of images in the dataset with different noise conditions.

Over 1000 photographs of grid pointer meters were utilized as datasets. These were collected at a substation, as shown in Figure 1. The datasets comprised images of meters under various working conditions, including shots taken at different angles and periods for the same meters. Data augmentation was also performed in varying degrees. These practices enriched the diversity of the dataset features, enabling the model to learn more generalized characteristics. It also helped reduce the risk of overfitting and improved overall model performance.

The main contributions of our work are as follows:

(1) In grid meter detection, PConv was introduced into the base module of YOLOv7 and it obtained a relatively optimal replacement structure through multiple experiments, reducing the model’s FLOPs and parameters.
(2) An adaptive calibration algorithm was proposed for pointer reading calculation, which exhibited a relatively high robustness and good detection accuracy.
(3) DyHead was integrated into YOLOv7, incorporating an attention mechanism that boosted the accuracy when detecting small target features.

The remainder of this paper is organized as follows: Section 2 provides the development of the critical technologies. Section 3 discusses the main technical improvements. The results of the experiments and ablation experiments are presented in Section 4. Finally, Section 5 and Section 6 summarize the paper, offering insights into the approach’s merits and outlining directions for future work.

## 2. Related Work

### 2.1. Real-Time Object Detection

Currently, there are two-stage detectors with a high detection accuracy, such as Fast R-CNN [12] and Faster R-CNN [13]. There are also high real-time one-stage object detectors, such as SSD [14] and the YOLO [15] series. Similarly, there are excellent semantic segmentation models like Mask R-CNN [5], which integrates the achievements of deep learning in computer vision.

The real-time performance becomes increasingly important as deep-learning vision models are implemented into industrial production. Typically, techniques such as pruning, quantization, and knowledge distillation [16] can be employed to reduce the model’s complexity. This can improve the inference speed and enhance the real-time performance. Additionally, improved convolution modules can also be applied to reduce the model’s parameters and FLOPs, such as depthwise convolution (DWConv) [17] and PConv. These modules can reduce the parameters, accelerate the model’s inference performance, and enhance the real-time capabilities to some extent.

### 2.2. Pointer Meter Reading

Past researchers have achieved substantial work in the grid meter reading field. Some researchers have employed traditional visual solutions in their work. They have utilized feature extraction methods to detect the pointer meter. Others have adopted deep learning, detecting meters through object detection and using semantic segmentation to delineate the regions of the pointers. Our team has accumulated a wealth of experience in meter reading. Past works have attempted to use image classification by detecting meters through sliding windows. Feature point detection has also been used to calibrate the dial to achieve meter readings. However, these methods could achieve a better real-time performance.

In this paper, PConv is introduced into the base module of the YOLOv7 backbone, replacing regular convolutions to reduce the computational load and increase the frames per second (FPS). Eisting work such as Sun et al. [18] has introduced PConv into the YOLO series networks. However, this work does not provide details on how the integration is performed, the number of replacements, and crucial aspects like the replacement positions. Therefore, our work experiments with various replacement approaches in the base module of the YOLOv7 backbone and identifies the most effective replacement through experimentation. Moreover, DyHead with an attention mechanism is added to the detection head of YOLOv7 to enhance the model’s performance in strip object recognition.

## 3. Methods

**Figure 2:**
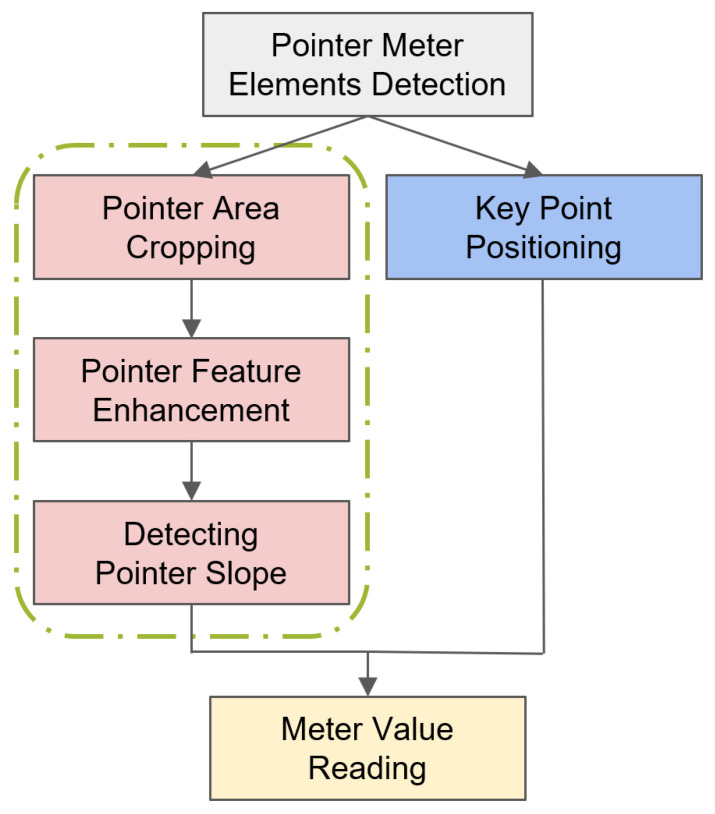
The entire algorithmic process.

The algorithmic process is illustrated in Figure 2. The meter image is initially captured and fed into the improved ${\mathrm{YOLOv7}}_{tiny}$ object detector to detect relevant elements in the meter dial. YOLOv7 is used to extract the positions of the short pointers and the regions corresponding to the left and right starting points of the instrument’s scale. The area of the detected pointer is cropped from object detection.

The detected pointer region and key point coordinates are input into the adaptive calibration program for pointer reading. As the linear features of the pointer are very distinct in the cropped image, the adaptive calibration algorithm is used to estimate the slope of the pointer relative to the dial center coordinate system.

The meter reading can be calculated through a proportional relationship using the pointer’s slope (k), dial center coordinate $\left(x_{c},y_{c}\right)$, and dual boundary location $\left(x_{b1},y_{b1}\right)$, $\left(x_{b2},y_{b2}\right)$.

The direction vector of the pointer is

(1) $$\vec{P}=\left(1,k\right)$$

The vector of the dual boundary location to the center is

(2) $$\begin{array}{c} {\vec{V}}_{left}=\left(x_{b1}-x_{c},y_{b1}-y_{c}\right)\\ {\vec{V}}_{right}=\left(x_{b2}-x_{c},y_{b2}-y_{c}\right) \end{array}$$

So, the angle from the pointer to the nearest boundary of the dial range can be calculated as

(3) $$Angle=\min(\arccos\left(\frac{\vec{P}\cdot {\vec{V}}_{left}}{|\vec{P}\left|\cdot \right|{\vec{V}}_{left}|}\right),\;\arccos\left(\frac{\vec{P}\cdot {\vec{V}}_{right}}{|\vec{P}\left|\cdot \right|{\vec{V}}_{right}|}\right))$$

Using this angle, the meter’s reading can be calculated through the proportional relationship.

The entire reading process is typically faster compared with using semantic segmentation, and it also exhibits a strong adaptability to different environments.

### 3.1. Data Augmentation

The pointer dataset for this paper was manually collected by team members at the substation, including meter images from various shooting angles, different periods, diverse lighting conditions, and varying pointer angles. Approximately 1000 images were annotated. The meter images were captured at distances ranging from 0.5 m to 2 m, showcasing a multi-scale characteristics.

Different levels of data augmentation techniques were incorporated into the meter datasets. Salt-and-pepper noise and random region blur were added to the meter images. Also, stains and old meters were simulated to enhance the network’s ability to generalize such patterns. Random region blur is a technique used to increase the variety of a dataset. It calculates a value by multiplying the shorter side of the image and a specific ratio. This value is used as the length and width of a square region. The square is randomly applied to a particular area of the image, and a large-kernel mean filter is employed within this region, resulting in an image with region blur. The number of squares is generally limited to three or fewer per image, and the ratio is typically selected from a range of 0.2 to 0.3. Experiments have shown this data augmentation technique has a specific positive impact on the detection of striped objects. Different angles and various aspect ratios of pointer meters were stimulated by applying perspective transformation and random rotation. Additionally, the diversity of the datasets was enhanced by randomly adjusting the images’ color space, brightness, and saturation within predefined ranges.

### 3.2. ELAN-PConv

**Figure 3:**
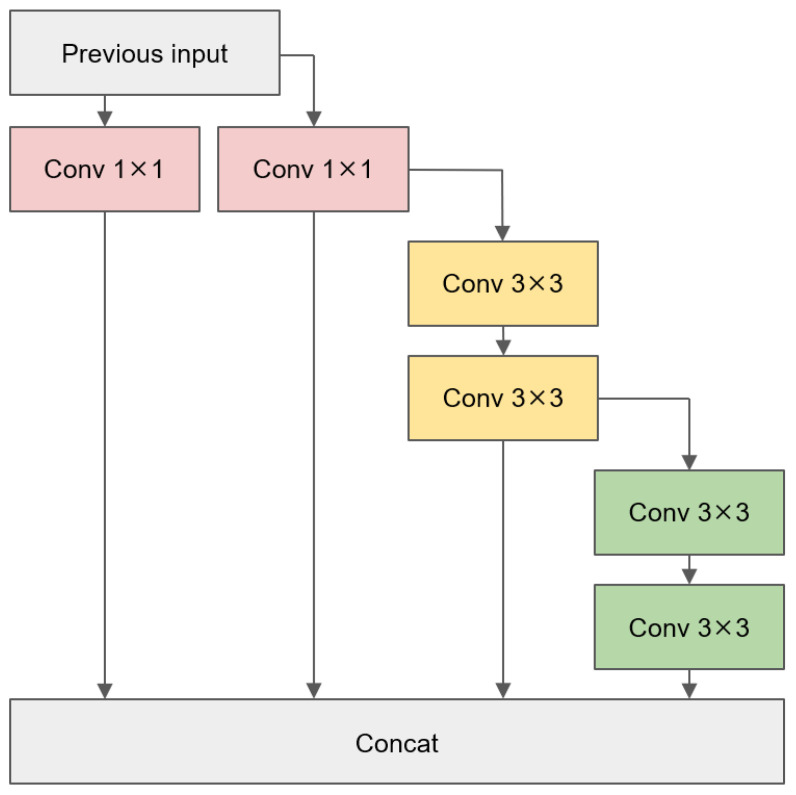
ELAN module structure in YOLOv7.

The original YOLOv7 base module was structured according to the ELAN module, as depicted in Figure 3. The entire backbone underwent hierarchical feature extraction through four layers of ELAN modules. The number of image channels progressed from 128 to 256, then to 512, and finally to 1024. 1×1 and 3×3 standard convolutions were utilized in each ELAN module. So, in each computation step, a large number of convolutional kernel operations were required. Taking the example of the ELAN module with an output channel of 256, a single computation between the two 3×3 convolutional layers utilized 4096 convolutional kernels. This undoubtedly imposed a significant computational burden, particularly for the edge devices. This resulted in a substantial computation overhead.

**Figure 4:**
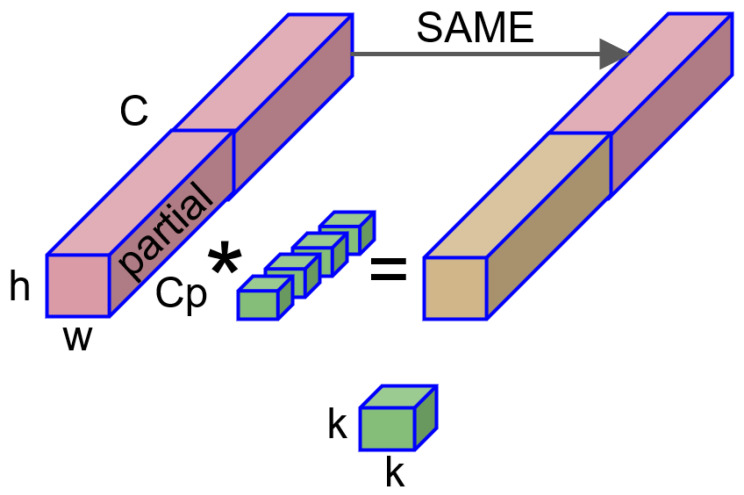
PConv computation diagram.

Inspired by the PConv proposed in FasterNet [10], Partial Convolution (PConv) represented a recent improvement in standard convolution. Its primary design goal was to reduce memory access and computational redundancy. The use of PConv was motivated by the observation that feature maps exhibited a high similarity across different channels, indicating a significant redundancy between channels. Therefore, leveraging the redundancy of the feature maps, PConv selectively applied standard convolution on a subset of channels while keeping the remaining channels unchanged, as shown in Figure 4. This reduced the computational complexity and contributed to realizing a fast and efficient neural network.

PConv was introduced at different positions in this paper to replace the standard convolutions in the ELAN modules. Validations were conducted to determine the best approach for introducing PConv in the Experiments Section.

PConv typically selects a certain proportion of channels for standard convolution, usually with *r* = $\frac{1}{4}$. When the channel numbers of input and output are the same, the FLOPs for using PConv are

(4) $$h\times w\times k^{2}\times c_{p}^{2}.$$

When $r=\frac{1}{4}$, PConv is simply $\frac{1}{16}$ of the standard convolution, it requires less memory accesses. The memory accesses for using PConv are

(5) $$h\times w\times 2c_{p}+k^{2}\times c_{p}^{2}\approx h\times w\times 2c_{p},$$

where *h* and *w* represent the feature map’s width and height, respectively, and *k* represents the size of the convolution kernel. $c_{p}$ refers to a subset of the feature channels.

When $r=\frac{1}{4}$, memory access is only $\frac{1}{4}$ of the standard convolution.

To integrate the feature information from different channels more effectively, PConv is typically followed by a Point-Wise Convolution (PWConv) [10] connection.

### 3.3. Change to DyHead

**Figure 5:**
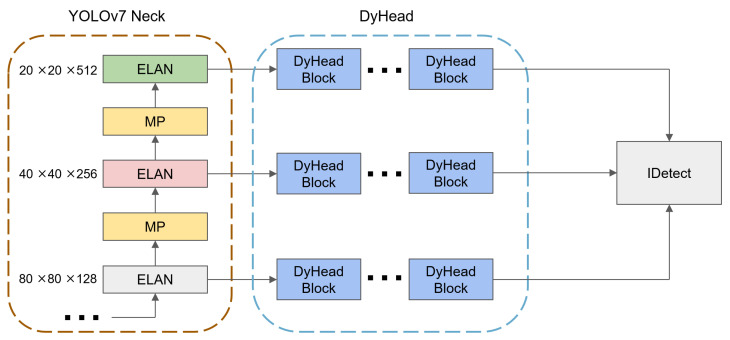
Dyhead added position in YOLOv7.

The IDetect module [9] of YOLOv7 was improved in this paper. IDetect is the prediction head of the YOLOv7 network, and is responsible for classification and regression. In the experiments of pointer detection in YOLOv7, the pointer scale and aspect ratio in each meter varied greatly. The performance was suboptimal for some relatively short and thin pointers. The features learned by the model were scattered, leading to a certain degree of deviation in the regression prediction boxes. So, DyHead was added before IDetect to enhance its perception of the scale and space, as shown in Figure 5.

**Figure 6:**
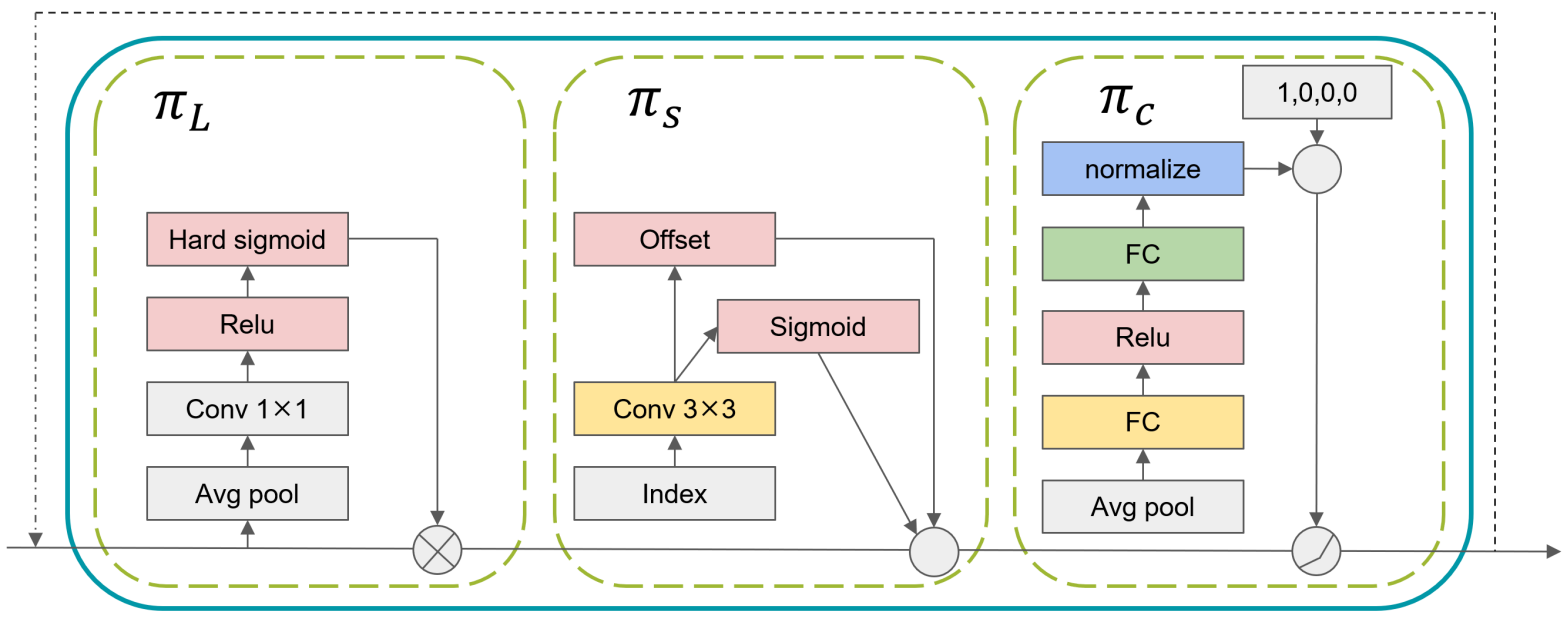
The internal structure of a single DyHead block.

Dynamic Head (DyHead) is a dynamic target detection head, as shown in Figure 6. It unified scale awareness, spatial awareness, and task-aware attention, constructing a self-attention mechanism. After feature extraction by the backbone network of YOLOv7, the obtained feature pyramids at three different scales were fed into DyHead. This involved utilizing a self-attention mechanism for spatial, scale, and task perception. The outputs were provided to IDetect for the classification and regression. This approach benefited the network by paying more attention to small objects or objects with significant aspect ratio differences. It enhanced the detection the performance of pointers.

**Figure 7:**
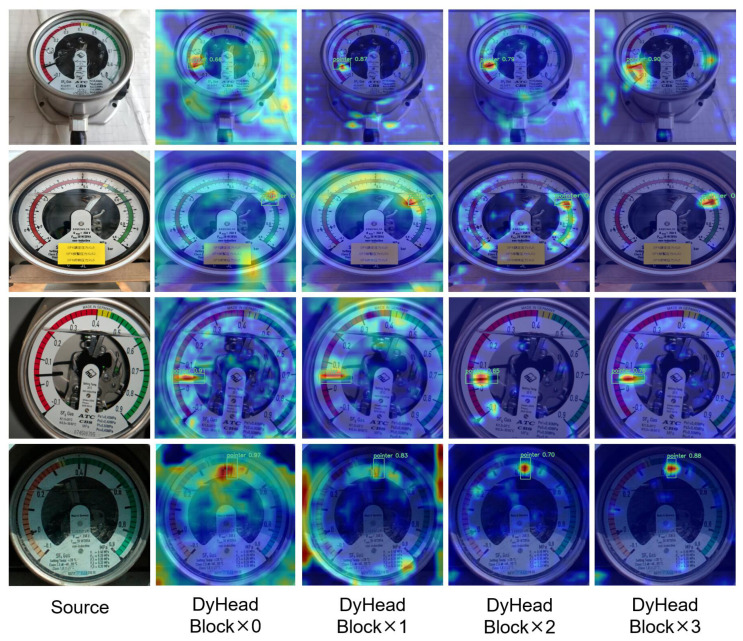
Stacking different numbers of DyHead blocks results in the heatmap.

The heatmap displayed in Figure 7 shows that DyHead’s multi-layer stacking enabled the feature layers to gradually converge onto the pointers without interference from the other dial components. Therefore, incorporating an attention mechanism into the model could enhance its ability to detect pointers with significant variations in spatial scales and aspect ratios, thereby improving its detection capability in complex environments.

### 3.4. Adaptive Calibration Pointer

**Figure 8:**
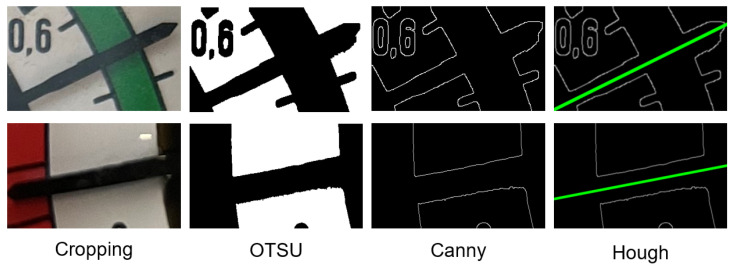
Process of extracting pointer features and fitting the slope.

After object detection, the region and key points of the pointer were input into the adaptive calibration program for meter reading. The detected key points mainly included the start and end positions on both sides of the meter range. This paper achieved this by cropping and duplicating a portion of the pointer region and applying Gaussian smoothing for noise reduction. The maximum interclass variance method (OTSU’s method) [19] was used to segment the pixels of the pointer. This is an adaptive threshold segmentation method. As short and thin pointers are typically in small regions on the dial face, the complexity of the dial face does not significantly impact the accurate segmentation of the pointer from the background. Moreover, OTSU’s method performs segmentation based on interclass variance and it is not influenced by image brightness and contrast. When obtaining the segmented image, we employed Canny edge detection [20], resulting in two distinct edges on the pointer. Hough line transform was applied to obtain the equations of these two lines. The complete process of extracting the pointer is schematized in Figure 8.

If the two lines are parallel, the program can arbitrarily select one as the fitted slope for the pointer. In the case of a sharp-tipped pointer, the average of the slopes of these two lines can be calculated and used as the fitted slope. The meter reading can be determined through proportional relationships by using the key points on both sides and the slope of the pointer.

## 4. Experiments

### 4.1. Experimental Setup

Experiments were conducted on Ubuntu 20.04. The software stack was configured with Python 3.8 and PyTorch 1.13.1. For hardware, training was performed on a Tesla P100 GPU. In the inference, the RTX 3060 Laptop was used to simulate the computational capabilities of the edge devices. The training code was based on the YOLOv7 project, which WongKinYiu opens sources on GitHub.

Regarding the model training parameter settings, Stochastic Gradient Descent (SGD) is the optimizer with an initial learning rate of 0.01, momentum of 0.937, and weight decay of 0.0005. The training was conducted on a batch size of 64 for 500 epochs of the dataset. Additionally, pre-trained weights were not utilized for a fair performance comparison between the different networks.

Mean Average Precision (mAP) was utilized as the accuracy evaluation metric. It is computed as the average precision (AP) average across all categories. Specifically, ${\mathrm{mAP}}_{50}^{val}$ calculates the average precision when the Intersection over Union (IoU) threshold is 50%.

(6) $$\mathrm{mAP}=\frac{1}{C}\sum_{c=1}^{C}{\mathrm{AP}}_{c}$$

where *C* represents the number of categories and *c* represents the specific category.

Using this evaluation metric helps better reflect the model’s accuracy in recognizing features.

### 4.2. Ablation Study

#### 4.2.1. PConv Replacement Position in ELAN

**Figure 9:**
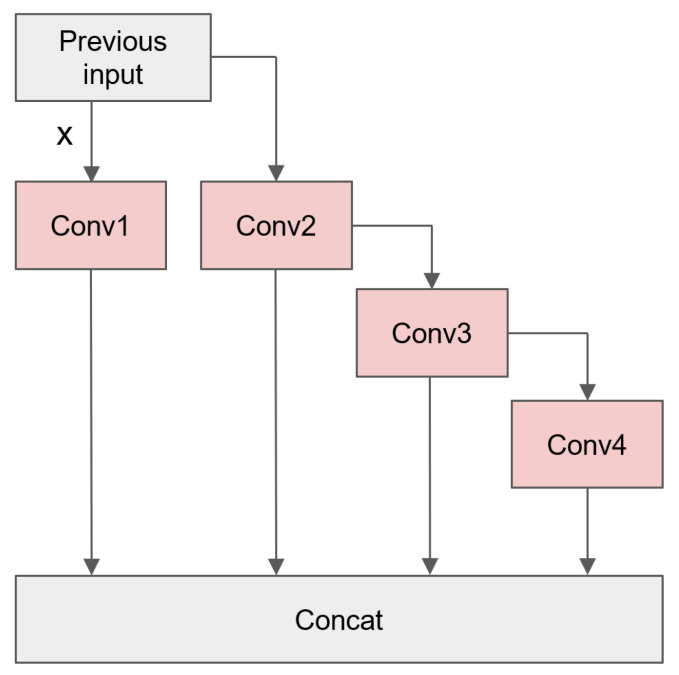
Location of the four Conv (standard convolutions) in the ELAN module of ${\mathrm{YOLOv7}}_{tiny}$.

Because of the characteristics of PConv, only a subset of channels underwent convolution, while the rest remained unchanged. Therefore, the position of replacing standard convolutions with PConv in the ELAN module was bound to affect the network’s performance. Figure 9 shows four replaceable convolution positions for the ELAN structure. Five replacement structures are shown in Table 1.

${\mathrm{YOLOv7}}_{tiny}$ + PConv-x refers to a different replacement position for PConv in the YOLOv7 ELAN module. Comparative experiments were conducted on different positions and quantities of PConv introduction and identified the most effective positional structure. The PConv module was configured to default to connecting a PWConv for feature fusion and channel transformation. All of the ${\mathrm{YOLOv7}}_{tiny}$ + PConv-x experiments were conducted directly on the original ${\mathrm{YOLOv7}}_{tiny}$ model.

Through experiments, ${\mathrm{YOLOv7}}_{tiny}$ + PConv-1 was found to have fewer parameters and FLOPs, as shown in Table 2. This is because all the Conv1-4 were replaced standard convolutions with PConv. This implies that only a subset of channels underwent convolution, resulting in fewer convolutional kernels. However, as the 3×3 convolution in ELAN was the primary feature extraction layer, replacing it may have led to insufficient feature extraction in some cases.

From Table 3, it is observed that ${\mathrm{YOLOv7}}_{tiny}$ + PConv-1 had the lowest ${\mathrm{mAP}}_{50}^{val}$, demonstrating insufficient feature extraction due to the reduction in the number of convolutional kernels. ${\mathrm{YOLOv7}}_{tiny}$ + PConv-4 exhibited the greatesr down inference time with a batch size 32, and it achieved a good ${\mathrm{mAP}}_{50:95}^{val}$. It was mainly replaced with PConv in the Conv2 and Conv4 positions.

#### 4.2.2. Module Ablation Study

Ablation experiments were conducted on the selected improved modules, as shown in Table 4. ${\mathrm{YOLOv7}}_{tiny}$-based PConv-1 had the most minor net parameters, and ${\mathrm{YOLOv7}}_{tiny}$-based PConv-4 had the relatively best ${\mathrm{mAP}}_{50:95}^{val}$. So, experiments were conducted by adding Dyhead to the ${\mathrm{YOLOv7}}_{tiny}$ + PConv-1 and ${\mathrm{YOLOv7}}_{tiny}$ + PConv-4 to determine which structure was more suitable.

From the experimental results, it can be observed that ${\mathrm{YOLOv7}}_{tiny}$ + PConv-4 + DyHead demonstrated a better performance than the other improved models. Despite the increase in parameters with the addition of DyHead, there was a reduction in FLOPs. The mAP also reached a relatively superior level.

#### 4.2.3. Meter Reading Comparison

**Figure 10:**
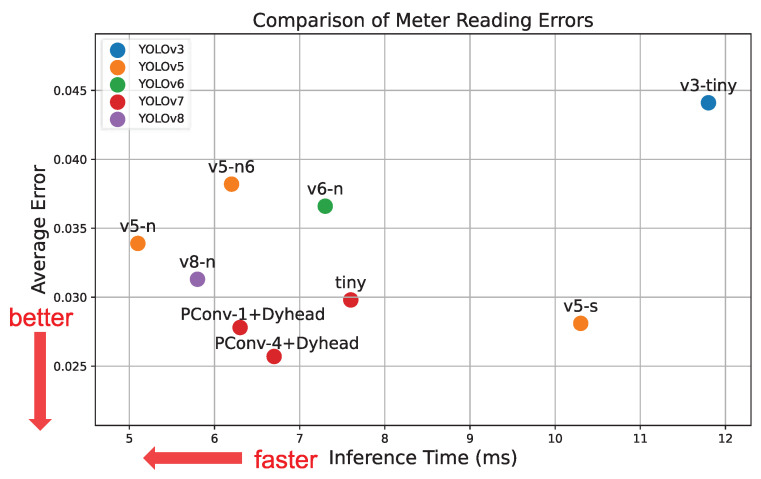
Comparison of meter reading with different models for feature extraction.

The meter reading errors using different YOLO models were compared. The pointer and keypoint features extracted by each YOLO model were fed into the reading program to calculate the meter value. The error was calculated by comparing the predicted meter values with the ground truth. This experiment processed all images in the test set to obtain each model’s average error and inference speed, as shown in Figure 10.

The models that were closer to the bottom-left corner were considered better. Compared with the YOLOv8n model, the original YOLOv7-tiny model had a higher accuracy, while YOLOv8n was faster. However, through field testing, our team concluded that accuracy was of a higher priority at the same parameter level. Therefore, we chose YOLOv7 as the base model.

## 5. Discussion

${\mathrm{YOLOv7}}_{tiny}$ + PConv-4 + DyHead was validated as being more suitable for meter datasets, providing a relatively optimal improvement structure through multiple experiments.

The improved network achieved a relatively optimal performance in mAP values compared with the other networks, as shown in Table 5. The ${\mathrm{mAP}}_{50}^{val}$ reached 97.87% and ${\mathrm{mAP}}_{50:95}^{val}$ reached 62.67%. Meanwhile, the parameters and FLOPs were maintained at a low level. This demonstrates the effectiveness of reducing the network volume through PConv and enhancing the feature perception with DyHead for meter detection.

## 6. Conclusions

Nowadays, numerous methods in grid meter reading are being used. However, these methods could be better in terms of both real-time performance and accuracy. This paper enhanced the speed and accuracy of grid meter detection in the improved YOLOv7 model. The ELAN module replaced the Standard convolutions with PConv to reduce the network’s parameters and FLOPs, enhancing the real-time inference on the edge devices. In addition, an attention mechanism was introduced by incorporating the DyHead Block into the neck output of YOLOv7 IDetect. It improved the network’s recognition of pointers and enhanced the meter detection accuracy. The ${\mathrm{mAP}}_{50}^{val}$ reached 97.87% and the ${\mathrm{mAP}}_{50:95}^{val}$ reached 62.67% in the improved model. The improved model took 6.4 ms to infer a single image, achieving a frames-per-second (FPS) rate of 108. It exhibited an excellent performance in terms of both accuracy and real-time capabilities.

**Figure 11:**
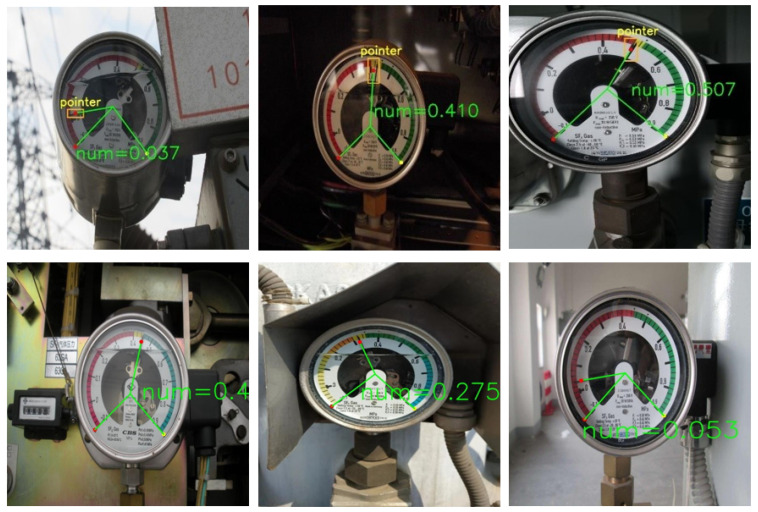
Actual industrial application examples of meter reading in the grid industry.

In future work, our team will further explore the challenges in grid meter detection. Lighter-weight networks will be investigated to expand the applicability of our work. We will explore how to achieve simple visual detection of meters on microcontrollers such as STM32H7 or MCX N Series. Our team will continue to advance the construction of industrial intelligence in the future, striving towards comprehensive intelligent manufacturing and intelligent inspection (Figure 11).

## Figures and Tables

**Table 1:** PConv replacement position (“✓” means this Conv in Figure 9 will be replaced).

Model	Conv1	Conv2	Conv3	Conv4
${\mathrm{YOLOv7}}_{tiny}$ + PConv-1	✓	✓	✓	✓
YOLOv7${\mathrm{YOLOv7}}_{tiny}$ + PConv-2	✓	✓		
${\mathrm{YOLOv7}}_{tiny}$ + PConv-3			✓	✓
${\mathrm{YOLOv7}}_{tiny}$ + PConv-4		✓		✓
${\mathrm{YOLOv7}}_{tiny}$ + PConv-5		✓	✓	✓

**Table 2:** Comparison of model complexity based on different PConv replacements.

Model	${\mathrm{Params}}_{\left(\mathrm{Net}\right)}$	${\mathrm{Params}}_{\left(\mathrm{Block}\right)}$	FLOPs
${\mathrm{YOLOv7}}_{tiny}$	6.02 M	1.31 M	13.2 G
${\mathrm{YOLOv7}}_{tiny}$ + PConv-1	**4.73 M**	0.41 M	10.5 G
${\mathrm{YOLOv7}}_{tiny}$ + PConv-2	6.12 M	1.38 M	13.6 G
${\mathrm{YOLOv7}}_{tiny}$ + PConv-3	4.83 M	0.33 M	10.1 G
${\mathrm{YOLOv7}}_{tiny}$ + PConv-4	5.35 M	0.86 M	11.9 G
${\mathrm{YOLOv7}}_{tiny}$ + PConv-5	4.78 M	0.37 M	10.3 G

**Table 3:** Performance comparison based on different PConv replacements.

Model	${\mathrm{Infer}}_{\left(b32\right)}$	FPS	${\mathrm{mAP}}_{50}^{\mathrm{val}}$	${\mathrm{mAP}}_{50:95}^{\mathrm{val}}$
${\mathrm{YOLOv7}}_{tiny}$	7.6 ms	104	97.13%	60.33%
${\mathrm{YOLOv7}}_{tiny}$ + PConv-1	6.2 ms	109	97.09%	59.61%
${\mathrm{YOLOv7}}_{tiny}$ + PConv-2	5.9 ms	114	98.12%	59.23%
${\mathrm{YOLOv7}}_{tiny}$ + PConv-3	7.1 ms	104	98.27%	59.05%
${\mathrm{YOLOv7}}_{tiny}$ + PConv-4	6.5 ms	108	97.75%	**60.48%**
${\mathrm{YOLOv7}}_{tiny}$ + PConv-5	6.1 ms	110	97.55%	59.34%

**Table 4:** Ablation experiments of the network improvement modules.

Model	Params	FLOPs	FPS	${\mathrm{mAP}}_{50}^{\mathrm{val}}$	${\mathrm{mAP}}_{50:95}^{\mathrm{val}}$
${\mathrm{YOLOv7}}_{tiny}$	6.02 M	13.2 G	104	97.13%	60.33%
${\mathrm{YOLOv7}}_{tiny}$ + Dyhead	5.96 M	13.0 G	106	97.83%	62.43%
${\mathrm{YOLOv7}}_{tiny}$ + Pconv-1	4.73 M	10.5 G	109	97.09%	59.61%
${\mathrm{YOLOv7}}_{tiny}$ + Pconv-4	5.35 M	11.9 G	108	97.75%	60.48%
${\mathrm{YOLOv7}}_{tiny}$ + Pconv-1 + Dyhead	4.77 M	10.5 G	110	97.80%	60.96%
${\mathrm{YOLOv7}}_{tiny}$ + Pconv-4 + Dyhead	5.37 M	11.8 G	108	**97.87%**	**62.67%**

**Table 5:** Comparison of real-time model performances.

Model	Params	FLOPs	${\mathrm{mAP}}_{50}^{\mathrm{val}}$	${\mathrm{mAP}}_{50:95}^{\mathrm{val}}$
${\mathrm{YOLOv7}}_{tiny}$	6.02 M	13.2 G	97.13%	60.33%
${\mathrm{YOLOv7}}_{tiny}$ + Pconv-1 + Dyhead	4.77 M	10.5 G	97.80%	60.96%
${\mathrm{YOLOv7}}_{tiny}$ + Pconv-4 + Dyhead	5.37 M	11.8 G	**97.87%**	**62.67%**
${\mathrm{YOLOv7}}_{tiny}$	12.1 M	19.0 G	95.38%	55.78%
YOLOv5n	1.7 M	4.2 G	96.62%	59.32%
YOLOv5n6	3.1 M	4.3 G	96.72%	58.94%
YOLOv5s	7.2 M	17.0 G	97.18%	59.35%
YOLOv6n	4.2 M	11.9 G	96.77%	59.51%
YOLOv8n	3.0 M	8.2 G	96.51%	59.73%

## Data Availability

The main code can be accessed from GitHub: https://github.com/Flora233333/Pointer-Reading-based-on-YOLO (accessed on 20 May 2024).
